# Establishment and characterization of a new intestinal-type ampullary carcinoma cell line, DPC-X3

**DOI:** 10.1186/s12885-024-13340-0

**Published:** 2024-12-20

**Authors:** Changpeng Chai, Xin Miao, Yuanhui Su, Cheng Yu, Huan Tang, Lu Li, Zhengfeng Wang, Jianfeng Yi, Zhenzhen Ye, Long Miao, Hui Zhang, Zhao Hu, Luyang Chen, Keren Wu, Ning Li, Linpei Wang, Wence Zhou, Hao Xu

**Affiliations:** 1 The Fourth Department of General Surgery, The First Hospital of Lanzhou University, Lanzhou, 730000 China; 2 The Second Clinical Medical College, Lanzhou University, Lanzhou, 730000 China; 3 Department of Nephrology, The First Affiliated Hospital of Zhejiang Chinese Medical University, Zhejiang Provincial Hospital of Chinese Medicine, Hangzhou, 310006 China; 4 The First School of Clinical Medicine, Zhejiang Chinese Medical University, Hangzhou, 310006 China; 5 The First Clinical Medical College, Lanzhou University, Lanzhou, 730000 China; 6 Department of Surgery, The First School of Clinical Medicine of Gansu University of Chinese Medicine, Lanzhou, 730000 China; 7 Department of General Surgery, Lanzhou University Second Hospital, Lanzhou, 730000 China; 8 Department of Hepatobiliary Surgery, The First Affiliated Hospital of Zhejiang Chinese Medical University, Zhejiang Provincial Hospital of Chinese Medicine, Hangzhou, 310006 China; 9 Department of Hepatobiliary and Pancreatic Surgery, The Second Affiliated Hospital of Fujian Medical University, Quanzhou, 362000 China; 10 Department of Hepatobiliary Surgery, The First Affiliated Hospital of Zhejiang Chinese Medical University, Zhejiang Provincial Hospital of Chinese Medicine, Hangzhou, 310006 Zhejiang China; 11 The First School of Clinical Medicine, Zhejiang Chinese Medical University, No. 54 Youdian Road, Shangcheng District, Hangzhou, Zhejiang 310006 China

**Keywords:** Ampullary carcinoma, Cell line establishment, Xenografted tumor, Short tandem repeat analysis, Drug resistance

## Abstract

Ampullary carcinoma (AC) of the intestinal type represents a distinct variant within the broader category of ampullary neoplasms. The scarcity of pertinent cellular models has constrained investigations centered on this particular malignancy. This research effectively generated a cell line (CL) of intestinal-type AC (DPC-X3). This newly developed CL has been continuously cultured for 1 year and has demonstrated stable passaging exceeding 60 generations. Morphologically, DPC-X3 exhibited characteristic attributes of an epithelial tumor. The cell proliferation rate of DPC-X3 exhibited a doubling interval of 79 h. Short tandem repeat (STR) analysis validated the high consistency between DPC-X3 and the patient’s primary tumor. Characteristically, DPC-X3 displayed sub diploid karyotypes, primarily featuring 44, XY inv (9), -18, -20, -22, and + mar. Under suspension culture conditions, DPC-X3 could efficiently form organoids, and DPC-X3 cells inoculated subcutaneously into NXG mice could form transplanted tumors. Drug susceptibility assays demonstrated that DPC-X3 resisted paclitaxel, oxaliplatin, 5-fluorouracil(5-FU), and gemcitabine. Immunohistochemical (IHC) evaluation revealed affirmative reactivity for CK7 and CK20 within DPC-X3 cells, while CDX2 exhibited no detectable expression. E-cadherin and Vimentin demonstrated positive immunoreactivity, whereas CEA and CA19-9 displayed faint positivity. The Ki-67 proliferation index was determined to be approximately 40%. DPC-X3 presents a valuable experimental platform for elucidating the pathogenesis of intestinal-type AC and can serve as a driver for drug development efforts.

## Introduction

Although rare, ampullary carcinoma (AC) has exhibited a steady rise in its occurrence rate in recent times. It originates from the intricate junction of the pancreatic duct, bile duct, and duodenum, and its precise oncogenesis and disease development are still not fully understood [1–3]. Initially classified by Kimura et al. into intestinal-type and pancreaticobiliary-type based on histological features, AC was later redefined by the World Health Organization in 2010. The updated pathological standards identified three distinct histological categories founded on morphological and IHC features: intestinal-type, pancreaticobiliary-type, and mixed-type [4–6].

Pancreaticoduodenectomy is the main treatment for ampullary cancer, with approximately 50% of cases having the possibility of surgical resection, followed by adjuvant treatment [7, 8]. Although the prognosis for intestinal-type AC is comparatively better than that for pancreaticobiliary and mixed types, the optimal value and regimen of adjuvant chemotherapy for intestinal-type AC remain uncertain [4, 9].

Therefore, understanding the pathogenesis of intestinal-type ampullary cancer is crucial to unveil new drugs and treatment methodologies for ampullary cancer, ultimately enhancing patient survival prospects.

Currently, only nine existing cell lines (CLs) globally are available for in vitro research on AC, with merely one CL representing intestinal-type AC, namely SNU869 [10, 11]. Recognizing the diversity in tumor etiology, tumor heterogeneity, and race-related genetic variations [12–15], establishing a diverse array of ampullary cancer CLs is crucial for advancing fundamental studies and pharmaceutical innovations in this field.

This research effectively developed a stable CL designated DPC-X3, representing intestinal-type ampullary cancer. This CL originated from tumor tissue surgically extracted from patients with ampullary cancer, providing a valuable experimental model for studying intestinal-type ampullary malignancies.

## Methodologies and materials

### Tissue source

**Fig. 1:**
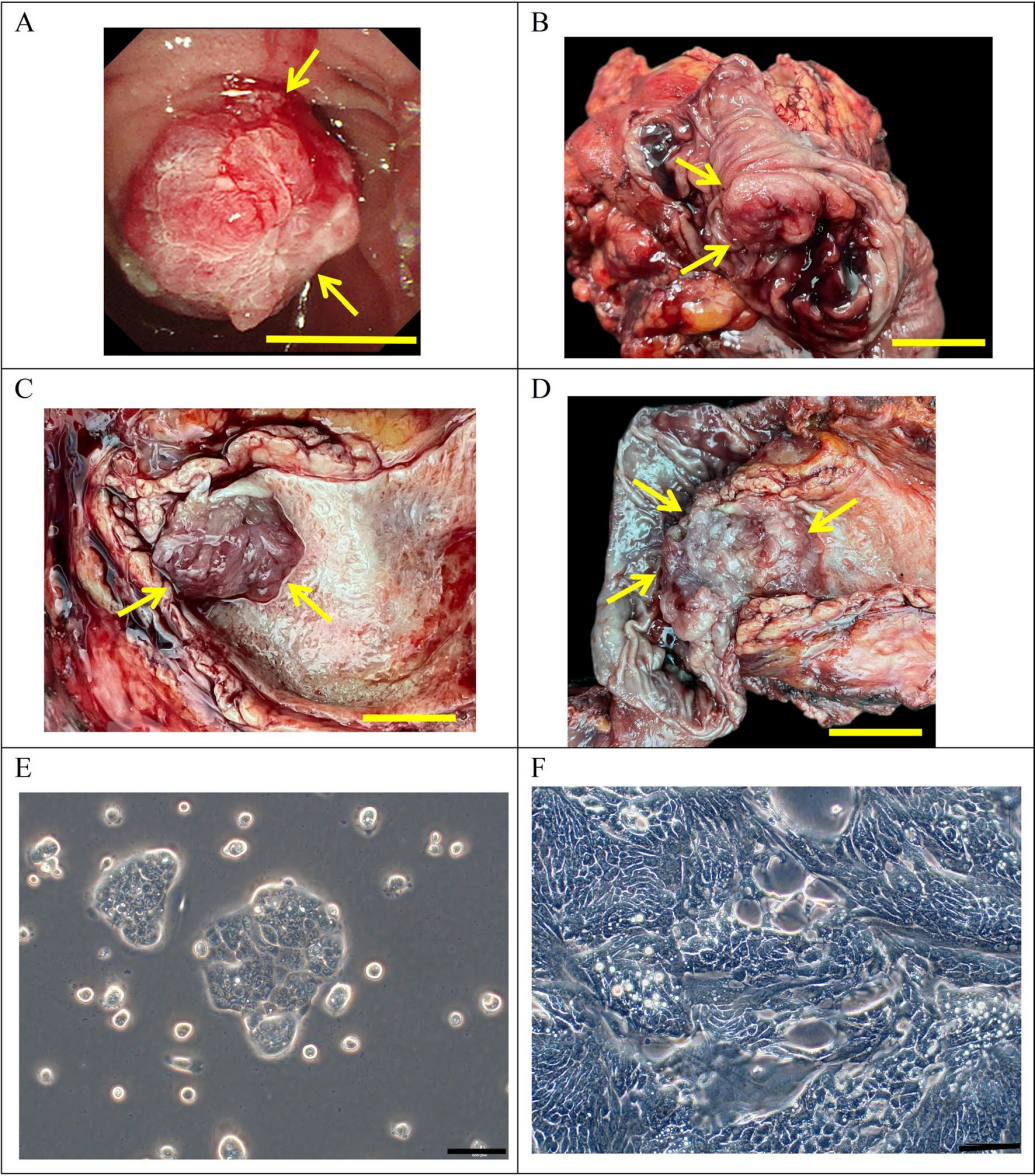
Clinical data and cell morphology. (**A**) Preoperative duodenoscopy findings of the patient depicting a tumor in the ampullary region (indicated by the arrow) (scale bar = 1 cm). (**B**–**D**) Gross examination of the postoperative specimen revealing a mass in the ampullary region (indicated by the arrow) (scale bar = 1 cm). (**E**–**F**) Microscopic examination showing DPC-X3 cell morphology under a light microscope (scale bar = 100 μm)

The subject, a 72-year-old man, reported no prior tobacco use, alcohol, or hepatitis B infection. His CEA level was 1.4 ng/mL (normal range: 0-5.2 ng/mL), AFP level was 4.6 U/mL (normal range: 0-5.8 U/mL), and CA 19 − 9 level was 38.3 U/mL (normal range: 0–35 U/mL; Table 1). Duodenoscopy examination indicated the presence of a tumor in the ampullary region (Fig. 1A). He did not undergo radiotherapy or chemotherapy before undergoing pancreaticoduodenectomy at Lanzhou University Second Hospital on August 29, 2022. Surgical gross views of the postoperative specimens revealed an ampulla with a 2 × 1.5 cm new organism (Fig. 1B-D). Samples were collected from the main site of infection for initial and subsequent cultivation examination.

**Table 1:** Clinical data of the included patient

Cell line	Patient age/ethicity	Gender	Histopathology/differentiation	Tumor size(cm)	Prior therapy	Culture date	Serum		
							AFP IU/ml (0-5.8)	CEA ng/ml (0-5.2)	CA19-9 U/ml (0–35)
DPC-X3	72/Asian	Male	Moderately	2 × 1.5	None	2022−8−29	4.6	1.4	38.3

This research was sanctioned by the Medical Ethics Committee of Lanzhou University Second Hospital (2023 A-381), with the participant granting informed agreement to participate.

### NXG mouse

The study utilized female NXG mice, 5–6 weeks of age, weighing 11–17 g. These rodents were obtained from Changzhou Cavens Laboratory Animal Co., Ltd. (Changzhou, China) and maintained in the specific pathogen-free facility at Lanzhou University’s Animal Experimental Center. The experimental design aimed to reduce animal distress. Prior to experimentation, mice were acclimated to laboratory settings (23℃, 12-h/12-h light/dark cycle, 50% humidity, unrestricted food, and water access) for 2 weeks. The animals were provided sterilized rodent feed without restriction, and their bedding, nourishment, and hydration were refreshed bi-daily. All protocols adhered to organizational and governmental standards for maintaining and utilizing laboratory fauna. The well-being and conduct of the subjects were monitored daily over four weeks. Mice were humanely euthanized when the xenograft tumor’s maximum diameter approached 1.5 cm or following 1 month of tumor development. Euthanasia was performed using an excessive dose of barbiturate (intravenous delivery, 150 mg/kg pentobarbital sodium), verified by the absence of heartbeat, respiration, and pupillary response.

The Medical Animal Experiment Ethics Committee of Lanzhou University Second Hospital provided ethical clearance for the animal research (D2023-318). All experiments complied with the ARRIVE guidelines and the Guidelines for the Care and Use of Laboratory Animals of China.

The techniques outlined in the following segments were comparable or equivalent to those utilized in earlier investigations [10, 16].

### Primary culture, cell purification, and CL establishment

The tumor specimen was immersed in aseptic phosphate-buffered saline (PBS; Gibco, Cat#10010023) and washed 3–5 times. After cutting it to the maximum extent possible, type II collagenase (Gibco, Cat#17101015) and Dispase II (Invitrogen, Cat#17105041) were added. The tissue was digested on a shaking table at 37 °C. The liquid above was procured after the limited enzymatic breakdown of the tissue samples. This fluid was strained using a 100-mesh sieve and centrifuged at 300 ×g for 3 min. The upper liquid was removed, and the residual material was reconstituted in PBS. After centrifugation at 300 ×g for 3 min, the sediment was mixed with full growth medium (RPMI-1640(BI, Cat#c3010-0500) + 10% fetal bovine serum [FBS](BI, Cat#04-002-1 A) + 1% penicillin-streptomycin (BI, Cat#03-031-1B)). This mixture was evenly seeded onto a six-well plate (NEST, Cat#703001), and the medium was replenished after 48 h. A sterile 1-mL pipette (KIRGEN) was used to scrape off fibroblasts repeatedly under direct microscope observation to remove fibroblasts from the primary culture process. Cell proliferation was routinely monitored using an optical microscope. From the third passage forward, cells underwent subculturing at a 1:2 proportion and were kept in a serum-free quick cell cryopreservation medium (Mei5 Biotechnology, Cat#MF699-01).

### Analysis of DNA short tandem repeat sequences

DPC-X3 cells in the exponential growth stage (EGS) (P10) were collected after trypsin digestion. These cells and the original neoplastic tissue underwent short tandem repeat (STR) profiling performed by Suzhou Genetic Testing Biotechnology Company to evaluate the relationship between the cultured cells and the source tumor specimen.

### Cell growth curve

DPC-X3 cells in the EGS (P25) were adjusted to a cell density of 1 × 10^4^/mL following trypsinization. Subsequently, 0.1 mL of the cell suspension was inoculated per well onto a 96-well plate. After cell attachment, CCK-8 reagent (Dojindo, Cat#ck04) was introduced for 4 consecutive days, with a reaction period of 2 h for each instance. The absorbance value at a wavelength of 450 nm was ascertained utilizing an enzyme-linked immunosorbent assay reader, and a calibration curve was constructed. The cell population doubling periods were calculated for multiple time intervals employing the “cell calculator++”tool [V. Roth MD, Doubling Time Calculator (2006), https://doubling-time.com/compute_more.php].

### Karyotyping

DPC-X3 cells in the EGS (P30) were exposed to 0.25-µg/mL colchicine for 6 h and throughout the night at 37 °C. Cells in metaphase were harvested, fixed using a methanol-acetic acid solution (3:1), stained utilizing Giemsa staining solution after trypsin digestion, and enumerated under microscopic examination. For karyotype evaluation, well-separated and adequately stained mitotic figures were chosen.

### Organoid culture from DPC-X3 cells

DPC-X3 cells (P33) underwent enzymatic digestion, centrifugation, and two PBS rinses in the EGS. The cells were then suspended in a growth medium (RPMI-1640 supplemented with 10% FBS and 1% penicillin-streptomycin, BI). The cell suspension (1,000 cells per well) was plated onto a non-adherent six-well plate (Corning, Cat#3471), with 2 mL of culture medium added for expansion. The development and quantity of organoids were routinely examined using a phase-contrast microscope.

### Scanning electron microscopy (SEM)

Cells in the logarithmic growth phase, harvested after 30 passages, were employed for SEM analysis. The cell-containing slides underwent washing with physiological saline, followed by fixation using a 4% glutaraldehyde solution (SPI-CHEM, USA). Subsequently, the samples were rinsed three times with phosphate buffer. Dehydration was conducted step-by-step using a gradient of tert-butanol (50%, 70%, 80%, 90%, and 100%) for 5 min each. The dried sample was placed into the JEOL JFD-320 freeze-drying apparatus, and upon equilibrating to ambient temperature, a conductive coating was affixed to the specimen mount utilizing a JEOL JFCC-160 ion sputtering device. The examinations were documented and micrographs were obtained employing a HITACHI Regulus 8100 scanning electron microscope.

### Transmission electron microscopy (TEM)

DPC-X3 cells (P30) in the EGS were subjected to enzymatic digestion, centrifugal separation, and fixation utilizing a suitable fixative for TEM analysis. The cellular samples were maintained and shipped at 4℃ before being processed for ultrathin sectioning and staining at Wuhan Servicebio Co., Ltd. (Wuhan, China). The prepared specimens were subsequently visualized utilizing a transmission electron microscope to examine intracellular components.

### Drug sensitivity test

Utilizing logarithmically growing DPC-X3 cells at passage 35 (P35), a single-cell suspension was prepared following trypsin digestion. Subsequently, 10,000 cells per well were inoculated and seeded into a 96-well plate. Upon cell adherence, the experimental group received varying concentrations of antitumor drugs: gemcitabine (600µmol/L, 150µmol/L, 30µmol/L, 6µmol/L, 1.5µmol/L, 0.3µmol/L, 0.06µmol/L, 0.015µmol/L, 0.003µmol/L, 0.0006µmol/L. plasma peak concentration: 19.01 µmol/L [17]), paclitaxel (50µmol/L, 25µmol/L, 12.5µmol/L, 2.5µmol/L, 0.5µmol/L, 0.1µmol/L, 0.025µmol/L, 0.005µmol/L, 0.001µmol/L. plasma peak concentration: 16.2 µmol/L [18]), oxaliplatin (400µmol/L, 200µmol/L, 100µmol/L, 40µmol/L, 20µmol/L, 10µmol/L, 5µmol/L, 2.5µmol/L, 1.25µmol/L, 0.625µmol/L, 0.3125µmol/L, 0.15625µmol/L. plasma peak concentration: 11.33 µmol/L [19]), and 5-fluorouracil [5-FU] (3840µmol/L, 960µmol/L, 240µmol/L, 60µmol/L, 15µmol/L, 3µmol/L, 0.6µmol/L, 0.15µmol/L, 0.03µmol/L, 0.006µmol/L. plasma peak concentration: 76.92 µmol/L [20]), while the control group received the corresponding drug solution. Following 72 h of drug treatment, the complete growth medium was substituted with 100 µL of a serum-free medium comprising 10% (v/v) CCK-8. Following a 2-hour incubation, the OD value was measured at 450 nm.

### Transplantation tumor formation experiment

Employing cells in the EGS at passage 33 (P33), the cellular concentration was regulated to 1 × 10^7^/mL following trypsin treatment and thoroughly homogenized. A pair of NXG mice were administered an inoculation of these cells (0.1 mL per animal) on the right scapular region and dorsal area. On the subsequent day, neoplasm development in the athymic rodents was monitored and documented. After 28 days, the animals were humanely sacrificed and examined to assess the progression of the xenografted tumor.

### IHC staining

Cells from the 42nd passage were enzymatically dissociated and plated on sanitized microscope slides for cultivation. Following a 48-hour incubation period, the adherent cells underwent a washing process with phosphate-buffered saline, immobilized using 4% paraformaldehyde solution for 15 min, allowed to dry in ambient air, and permeabilized with 0.5% Triton X-100 for 20 min. Sections of xenograft tumors and original neoplastic tissues were embedded in paraffin and subjected to overnight heating at 60 ℃.

The tissue samples underwent dewaxing, gradient alcohol hydration, and antigen retrieval utilizing the Autostainer Link 48 device from Dako. Afterwards, 3% hydrogen peroxide solution was added, followed by incubation at 37 ℃ for 15 min to inhibit peroxidase activity. Then, 100 µL of normal goat serum was dispensed dropwise, succeeded by a 15-minute incubation at 37 ℃ for blocking purposes.

Primary antibody (Fuzhou Maixin ready-to-use antibodies [CK7, Cat#MAB-0828; CK20, Cat#MAB-0834; CDX2, Cat#MAB-1056; Ki-67, Cat#MAB-0672; E-cadherin, Cat#MAB-0738; Vimentin, Cat#MAB-0735; CEA, Cat#MAB-0852; CA19-9, Cat#MAB-0778]) was introduced to the cells and kept at 37 ℃ for 1 h. The DAB staining kit (Dako, Cat#K3468) was utilized for chromogenic development, succeeded by washing the cells under flowing water for 5 min. After counterstaining with hematoxylin, the samples underwent dehydration and xylene clearing, and neutral resin was employed to seal the coverslip. Subsequently, the slides were examined under a microscope.

### Whole-genome resequencing (WGS) of DPC-X3 cells

Sequencing and data analysis were conducted at OE Biotech (Shanghai OE Biotech Co., Ltd). In short, genomic DNA was extracted from the DPC-X3 sample, and after passing the electrophoresis detection, the library was constructed. After the library construction was qualified, used a sequencer for dual end sequencing. After obtaining raw sequencing data from the sequencing machine, bioinformatics analysis was performed.

### Statistical analyses

All statistical analyses were performed using the SPSS 26.0 software (IBM, Armonk, NY, USA). Data were expressed as mean ± standard deviation (SD). Student’s t-tests and Analysis of Variance (ANOVA) were used for two-group and multi-group comparisons, respectively. A *p*-value less than 0.05 was considered statistically significant.

## Results

### Establishment of DPC-X3 CL

A stable and passaged ampulla cancer CL, DPC-X3, was successfully established by isolating primary cells from surgical tumor tissue and subsequent culturing. Under an optical microscope, DPC-X3 exhibited typical epithelial cell-like morphology with cell island–like adherent growth. These polygonal cells possess a large nucleus and prominent nucleoli (Fig. 1E and F). The cellular structure maintained stability even following multiple cycles of cryopreservation and revival.

### Analysis of DNA short tandem repeat sequences

The DNA typing outcomes verified that the two provided specimens stem from a single source, yielding a likelihood ratio (LR) of 2.4864 × 10^26^ (Table 2). This substantial LR strongly supports the derivation of the DPC-X3 CL, which originated from the identical subject as the primary neoplasm sample. Moreover, the genetic signature of DPC-X3 showed no correspondence with any current records in the ExPASy STR repository, validating its classification as a previously undescribed human AC CL.

**Table 2:** Short tandem repeat profile of DPC-X3 and tumor tissue

Loci	DPC-X3		Tumor tissue		Matching probability
Amelogenin	X	Y	X	Y	/
D3S1358	15	17	15	17	0.1540
D5S818	10	11	10	11	0.1287
D2S1338	18	22	18	22	0.0202
TPOX	8		8		0.2862
CSF1PO	10	12	10	12	0.1415
Penta D	11	13	11	13	0.0424
TH01	6	10	6	10	0.0086
vWA	16	18	16	18	0.0486
D7S820	10	11	10	11	0.1350
D21S11	30	31	30		/
Penta E	11	14	11	14	0.0306
D10S1248	15		15		0.0414
D8S1179	15		15		0.0256
D1S1656	13	15	13	15	0.0003
D18S51	14		13	14	/
D12S391	18	20	18	20	0.0733
D6S1043	14		14	19	/
D19S433	13	15	13	15	0.0244
D16S539	9	12	9	12	0.1283
D13S317	8		8		0.0484
FGA	22	25.2	22	25.2	0.0010
LR					2.4864 × 10^26^

### Cell growth curve

**Fig. 2:**
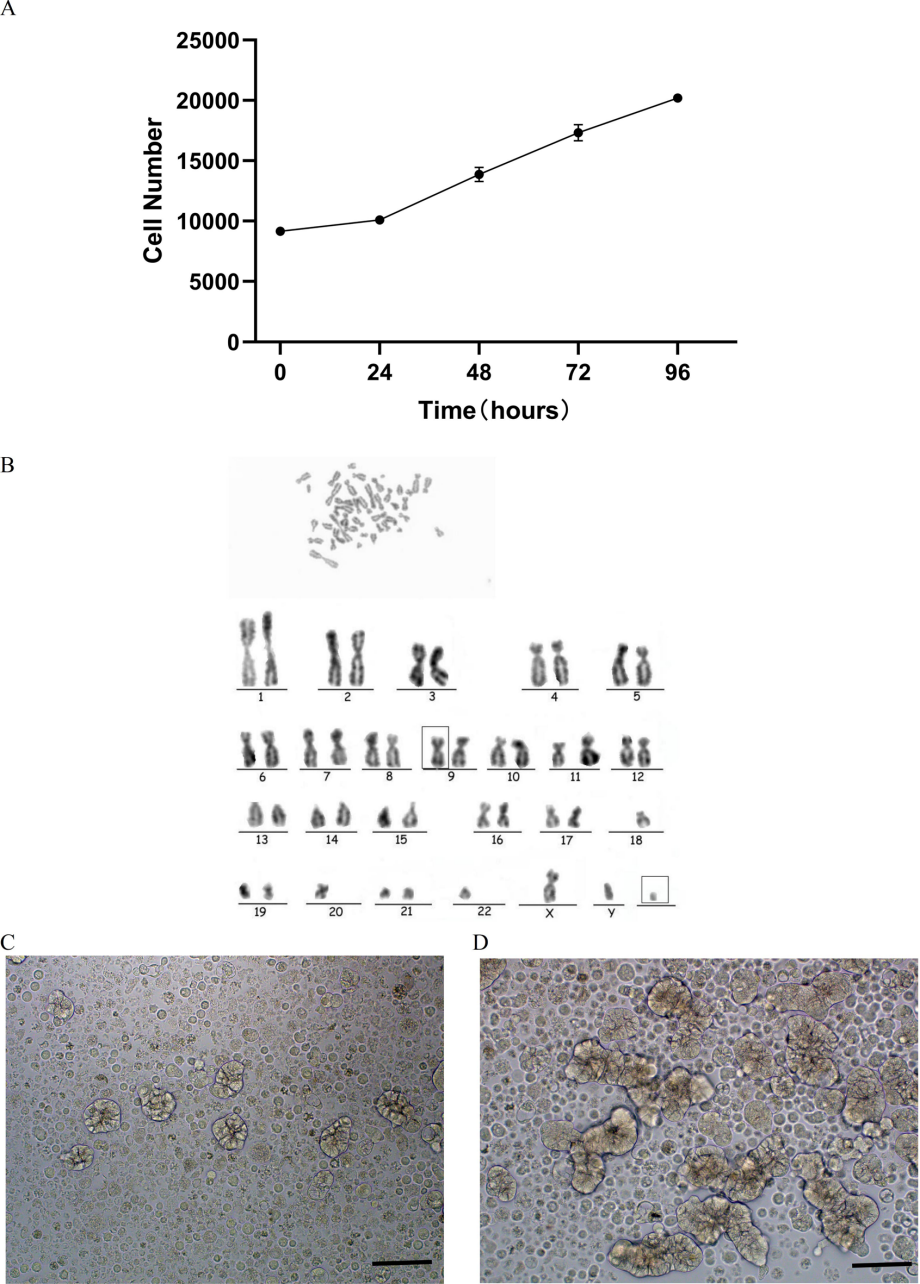
DPC-X3 cell characteristics. (**A**) Growth curve illustrating the doubling time of the DPC-X3 cell population was 79 h. (**B**) Karyotype analysis revealed that 80% of DPC-X3 cells were subdiploid and 20% were subtriploid, with representative karyotype shown as 44, XY inv (9), -18, -20, -22, +mar. (**C**) Morphology of DPC-X3 cell organoids after 4 days under suspension culture condition (scale bar = 50 μm). (**D**) Morphology of DPC-X3 cell organoids after 10 days under suspension culture condition (scale bar = 50 μm)

The proliferation rate of DPC-X3 cells was moderate, exhibiting steady growth in RPMI-1640 medium comprising 10% FBS. Utilizing the CCK-8 technique, the population doubling period of DPC-X3 cells was 79 h (Fig. 2A).

### Karyotyping

Karyotype analysis indicated that 80% of DPC-X3 cells were subdiploid and 20% were subtriploid, displaying chromosome count and morphology variations. Representative karyotypes were 44, XY inv (9), -18, -20, -22, +mar (Fig. 2B).

### Organoid culture

Upon inoculating DPC-X3 cells on an ultra-low attachment culture dish, robust cell proliferation was observed in a complete culture medium, forming glandular-like tumor organoids. Over time, these organoids grew larger, exhibiting increased complexity in glandular structures, and developed longer branching patterns (Fig. 2C and D).

### SEM and TEM

**Fig. 3:**
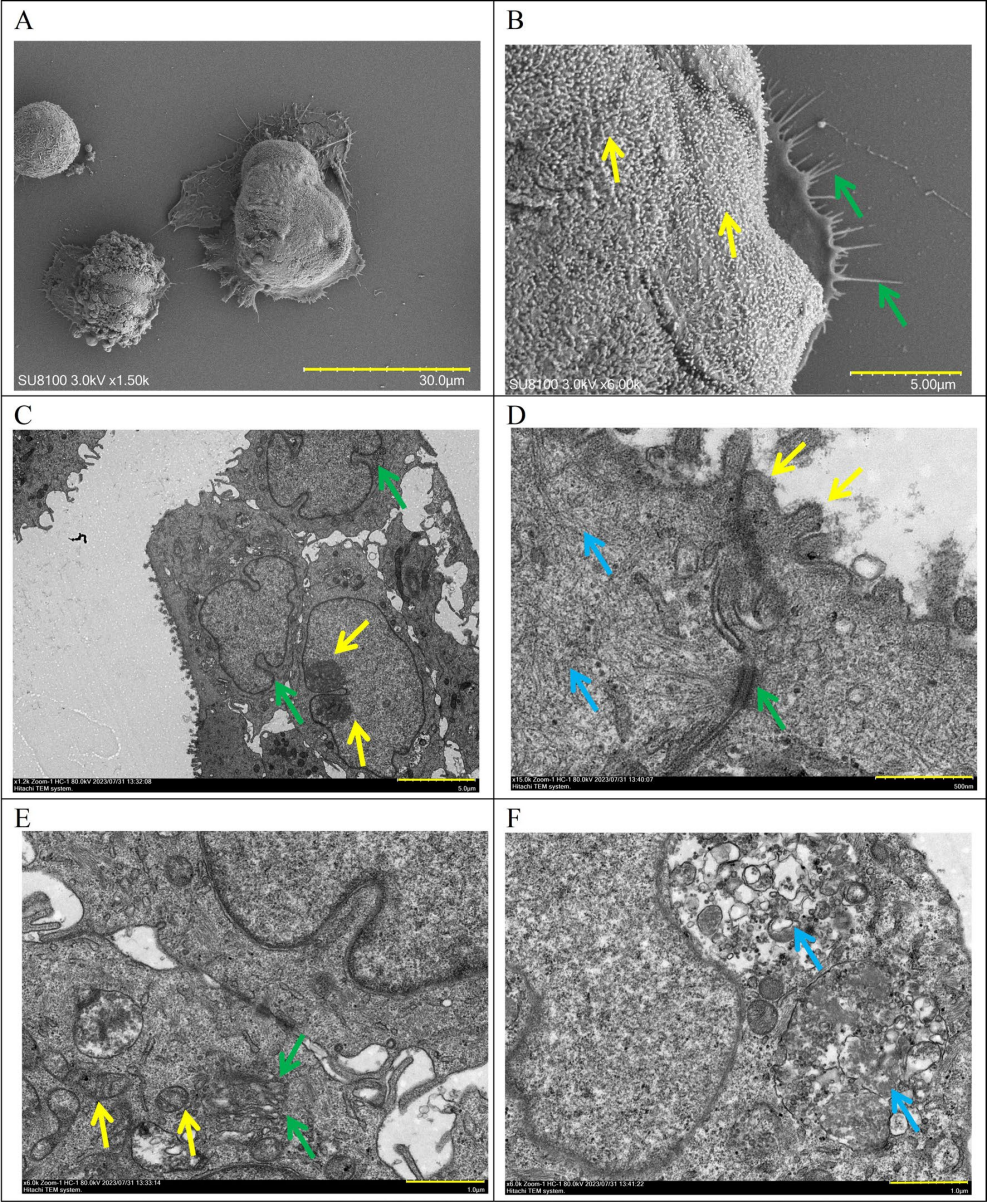
Ultrastructural examination of DPC-X3 cells. (**A**-**B**) DPC-X3 cells are spherical and have a relatively uniform morphology, with abundant microvilli on the cell surface (yellow arrow) and filamentous pseudopodia visible (green arrow) (**A**, scale bar = 30 μm; **B**, scale bar = 5 μm). (**C**) The nucleus of DPC-X3 has enlarged, the number of nucleoli has increased, the edges appear to be gathered (yellow arrow), the nuclear membrane has shrunk (green arrow), and the cytoplasm content has decreased (scale bar = 5 μm). (**D**) Microvilli can be seen on the cell surface (yellow arrow), with a desmosome structure (green arrow) and several tension fibrils inside the cell (blue arrow) (scale bar = 500 nm). (**E**) The number of intracellular organelles has decreased, mitochondria have swollen and vacuolized, and the number of cristae decreases (yellow arrow). A certain amount of Golgi apparatus (green arrow) appears in the cell (scale bar = 1 μm). (**F**) Lysosomes (blue arrow)rich in zymogen particles can be seen (scale bar = 1 μm)

Examination through SEM unveiled DPC-X3 cells exhibiting a spheroidal form, characterized by copious microvilli adorning the cellular exterior (indicated by yellow arrow) and a comparatively consistent morphological appearance, displaying thread-like pseudopodia (denoted by green arrow; Fig. 3A and B). TEM showcased enlarged nuclei in DPC-X3, increased nucleoli gathering toward the edges (yellow arrow), nuclear membrane shrinkage (green arrow), and reduced cytoplasm.

Microvilli could be observed on the cell surface (yellow arrow), and a desmosome structure (green arrow) and numerous tension fibrils (blue arrow) were observed inside the cell. The number of intracellular organelles decreased, showing swollen mitochondria with reduced cristae (yellow arrow). Golgi apparatus (green arrow) and zymogen particle-rich lysosomes (blue arrow) were observed (Fig. 3C-F).

### Drug sensitivity test

**Fig. 4:**
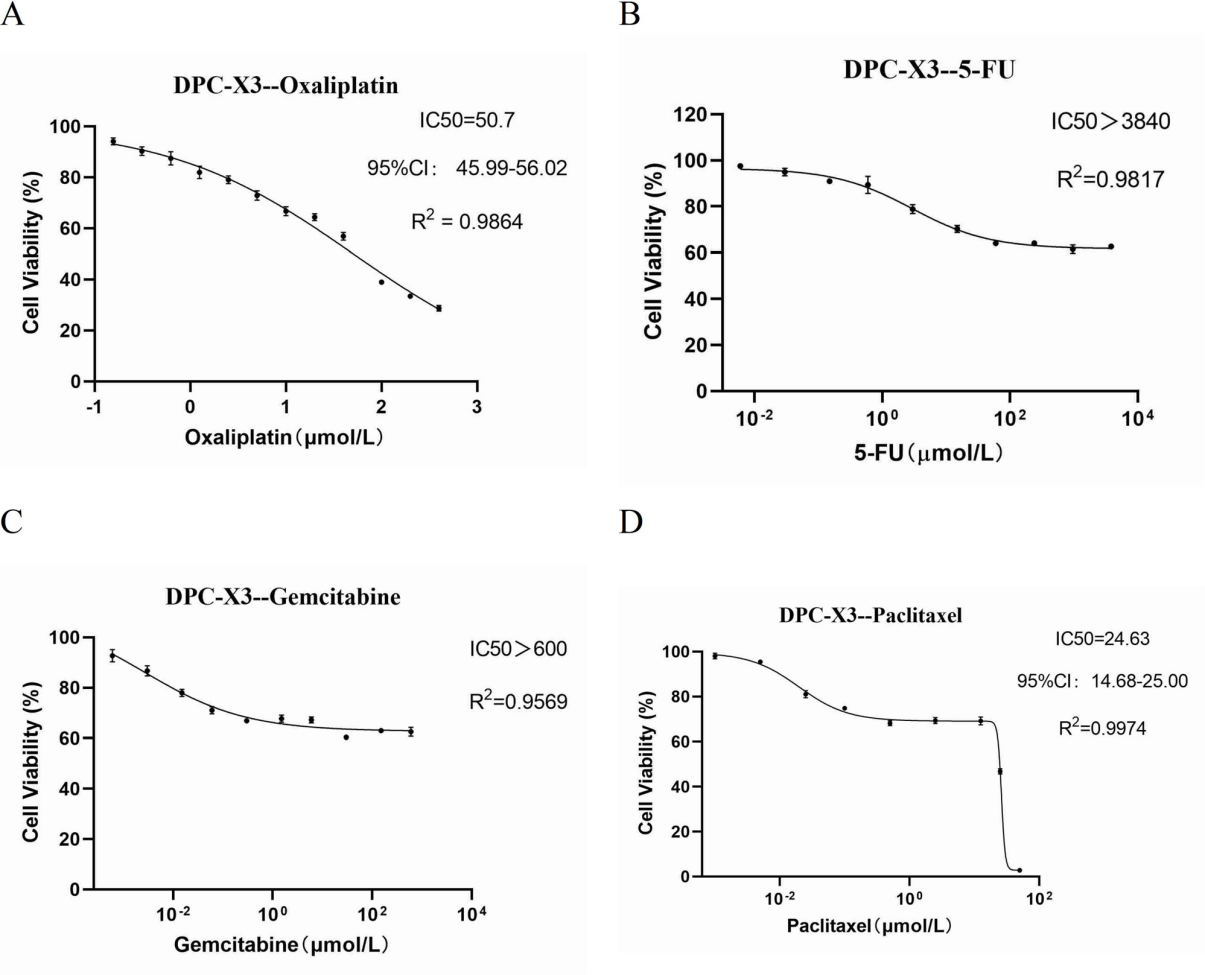
Assessment of anticancer drug sensitivity in DPC-X3 cells. DPC-X3 cells demonstrated resistance to oxaliplatin (**A**), fluorouracil (**B**), gemcitabine (**C**), and paclitaxel (**D**)

Chemotherapy drugs such as gemcitabine, paclitaxel, 5-FU, and oxaliplatin are frequently employed in treating gastrointestinal malignancies. The drug response assay demonstrated DPC-X3’s resistance to oxaliplatin (IC50 = 50.7 µmol/L); it exhibited high resistance to fluorouracil (IC50 > 3840 µmol/L) and gemcitabine (IC50 > 600 µmol/L), along with moderate resistance to paclitaxel (IC50 = 24.63 µmol/L; Fig. 4A-D).

### Transplantation tumor formation experiment

**Fig. 5:**
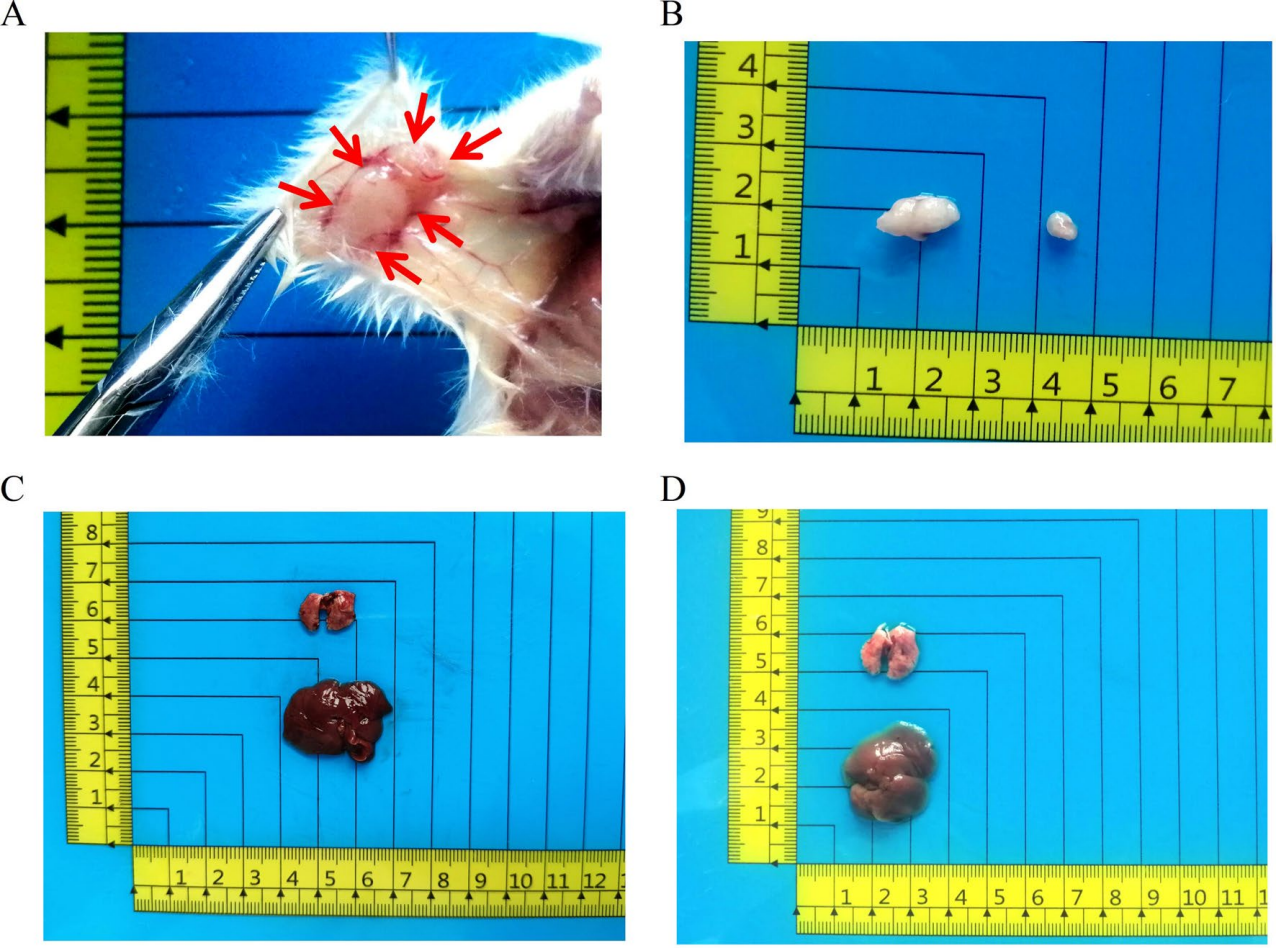
In vivo studies on xenograft tumor formation by DPC-X3 cells. (**A**-**B**) Subcutaneous transplantation of DPC-X3 cells in NXG mice resulted in notable tumor formation (red arrow), the tumor formation rate was 100%, and the tumor volume was 161.5 ± 111.5 mm^3^. (**C**-**D**) No metastatic lesions were observed in the liver and lung tissues of NXG mice 4 weeks after transplantation

To validate the capacity of DPC-X3 for generating transplanted tumors in vivo, 1 × 10^6^ DPC-X3 cells were subcutaneously inoculated into 2 NXG mice. The results indicated that DPC-X3 could form transplanted tumors in nude mice 4 weeks after subcutaneous inoculation, exhibiting a 100% tumor formation rate, the tumor volume was 161.5 ± 111.5 mm^3^ (Fig. 5A and B). Examination of the dissected mice revealed no metastatic sites in either the liver or lungs (Fig. 5C and D).

### H&E and immunohistochemical staining

**Fig. 6:**
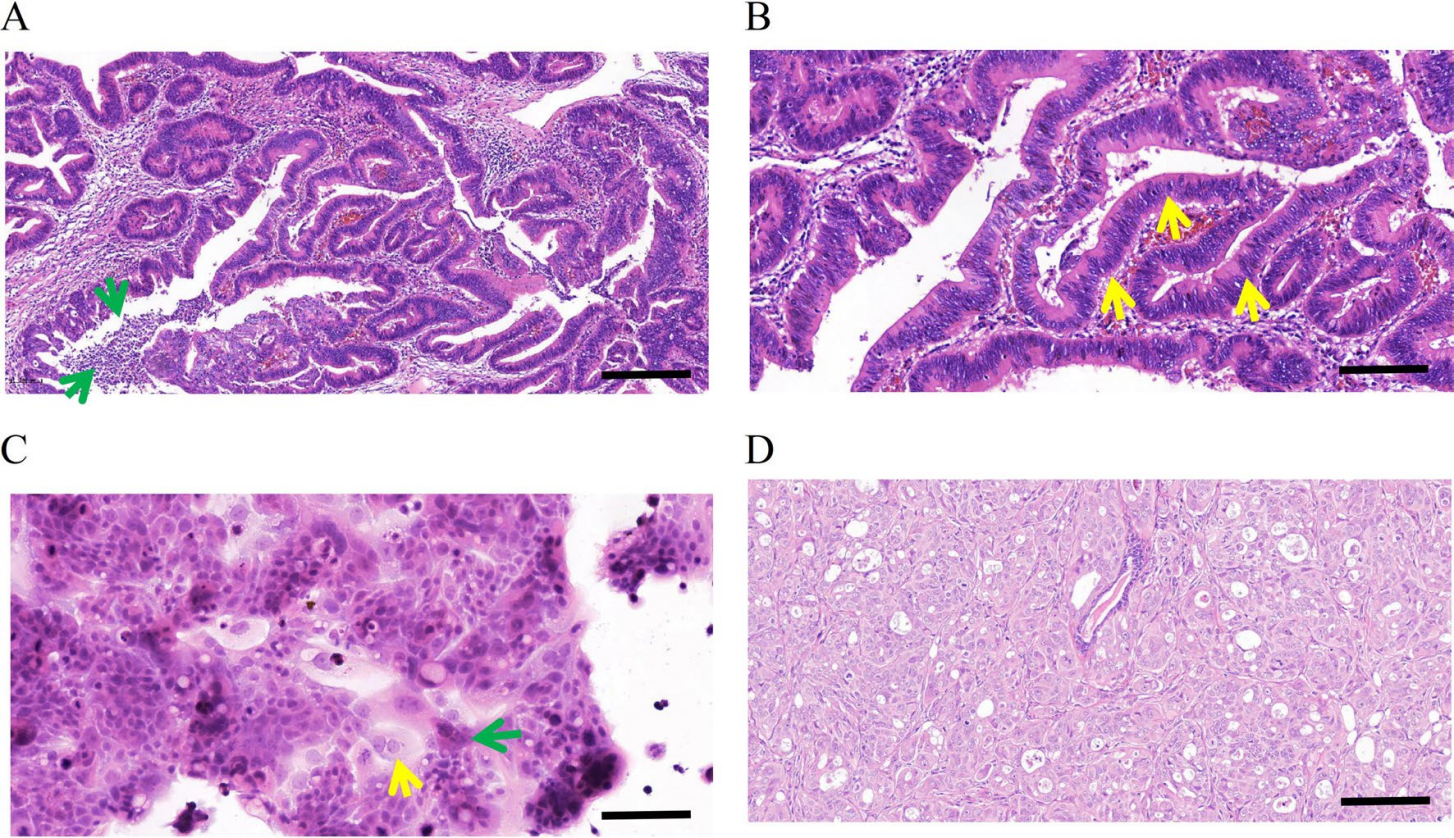
Hematoxylin-eosin (H&E) staining of the primary tumor, DPC-X3 cells, and xenograft tumor. The primary tumor was moderately differentiated. The tumor cells are arranged in an irregular glandular tubular pattern, large tubules lined by tall columnar cells with elongated, pseudostratified, hyperchromatic nuclei (yellow arrow). Luminal necrotic debris and acute inflammatory cells (green arrow) are also present. Typical structural characteristics are consistent with intestinal ampullary carcinoma (**A**, scale bar = 100 μm; **B**, scale bar = 50 μm). H&E staining showed that the morphology of DPC-X3 cells was relatively uniform, mainly polygonal, with an enlarged nucleus and evident nucleoli. The number of nucleoli increased, and the cytoplasm was less. A small number of multinucleated (yellow arrow) and megakaryocytes (green arrow)were visible (**C**, scale bar = 50 μm). H&E staining of the transplanted tumor shows irregular glandular structures formed by the cells, and its histological morphology was similar to that of the primary tumor (**D**). (scale bar = 50 μm)

The patient’s primary tumor was classified as moderately differentiated upon postoperative pathological examination. The neoplastic cells exhibited an atypical glandular tubular arrangement, characterized by expansive tubules bordered by elongated columnar epithelium with stretched, multilayered, deeply stained nuclei (yellow arrow). Intraluminal necrotic material and acute inflammatory infiltrates (green arrow) were also observed, aligning with the distinctive architectural features of intestinal-type AC (Fig. 6A and B).

H&E staining showed relatively uniform polygonal-shaped DPC-X3 cells, exhibiting enlarged nuclei, prominent nucleoli, and diminished cytoplasm. The sample displayed certain multinucleated cells (yellow arrow) and megakaryocytes (green arrow), presenting typical malignant tumor cell characteristics (Fig. 6C).

H&E examination of the grafted tumor exhibited atypical gland-like formations resembling those observed in the original malignancy (Fig. 6D).

**Fig. 7:**
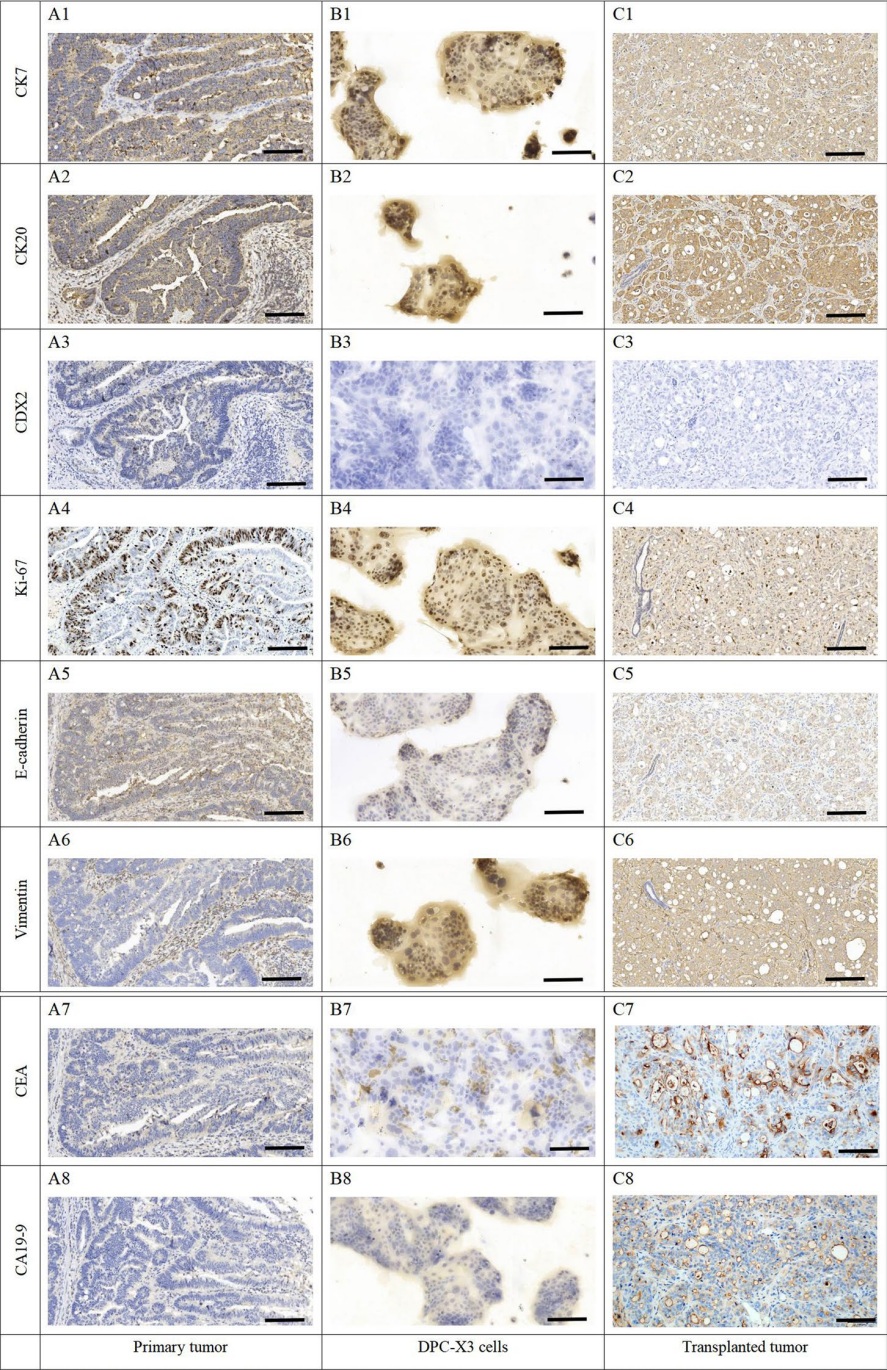
Immunohistochemical staining of primary tumor, DPC-X3 cells, and xenograft tumor. (**A1**), (**B1**), and (**C1**) CK7-positive staining of primary tumor (**A1**), DPC-X3 cells (**B1**), and xenograft tumor (**C1**). (**A2**), (**B2**), and (**C2**) CK20-positive staining of primary tumor (**A2**), DPC-X3 cells (**B2**), and xenograft tumor (**C2**). (**A3**), (**B3**), and (**C3**) CDX2-negative staining of primary tumor (**A3**), DPC-X3 cells (**B3**), and xenograft tumor (**C3**). (**A4**), (**B4**), and (**C4**) Quantitative analysis showed a 40% positive rate of Ki67 in primary tumor (**A4**), DPC-X3 cells (**B4**), and xenograft tumor (**C4**). (**A5**), (**B5**), and (**C5**) Positive expression of E-cadherin was observed in primary tumor (**A5**), DPC-X3 cells (**B5**), and xenograft tumor (**C5**). (**A6**), (**B6**), and (**C6**) Positive expression of Vimentin was observed in primary tumor (**A6**), DPC-X3 cells (**B6**), and xenograft tumor (**C6**). (**A7**), (**B7**), and (**C7**) CEA was focally positive expressed in primary tumor (**A7**), DPC-X3 cells (**B7**), and xenograft tumor (**C7**). (**A8**), (**B8**), and (**C8**) CA19-9 was weakly expressed in primary tumor (**A8**), DPC-X3 cells (**B8**), and xenograft tumor (**C8**). (scale bar = 50 μm)

IHC displayed affirmative staining for CK7 (Fig. 7A1-C1) and CK20 (Fig. 7A2-C2) in primary tumors, DPC-X3 cells, and transplanted tumors, while CDX2 (Fig. 7A3-C3) expression was negative, indicating a common origin. The Ki-67 (Fig. 7A4-C4) proportion at 40% aligned with rapid tumor cell proliferation. Low E-cadherin (Fig. 7A5-C5) expression and positive Vimentin (Fig. 7A6-C6) expression indicated strong epithelial-to-mesenchymal transition (EMT) characteristics in DPC-X3. Additionally, CEA (Fig. 7A7-C7) showed focal expression, while CA19-9 exhibited weak positivity (Fig. 7A8-C8).

### Mutation analysis of key driver genes

**Fig. 8:**
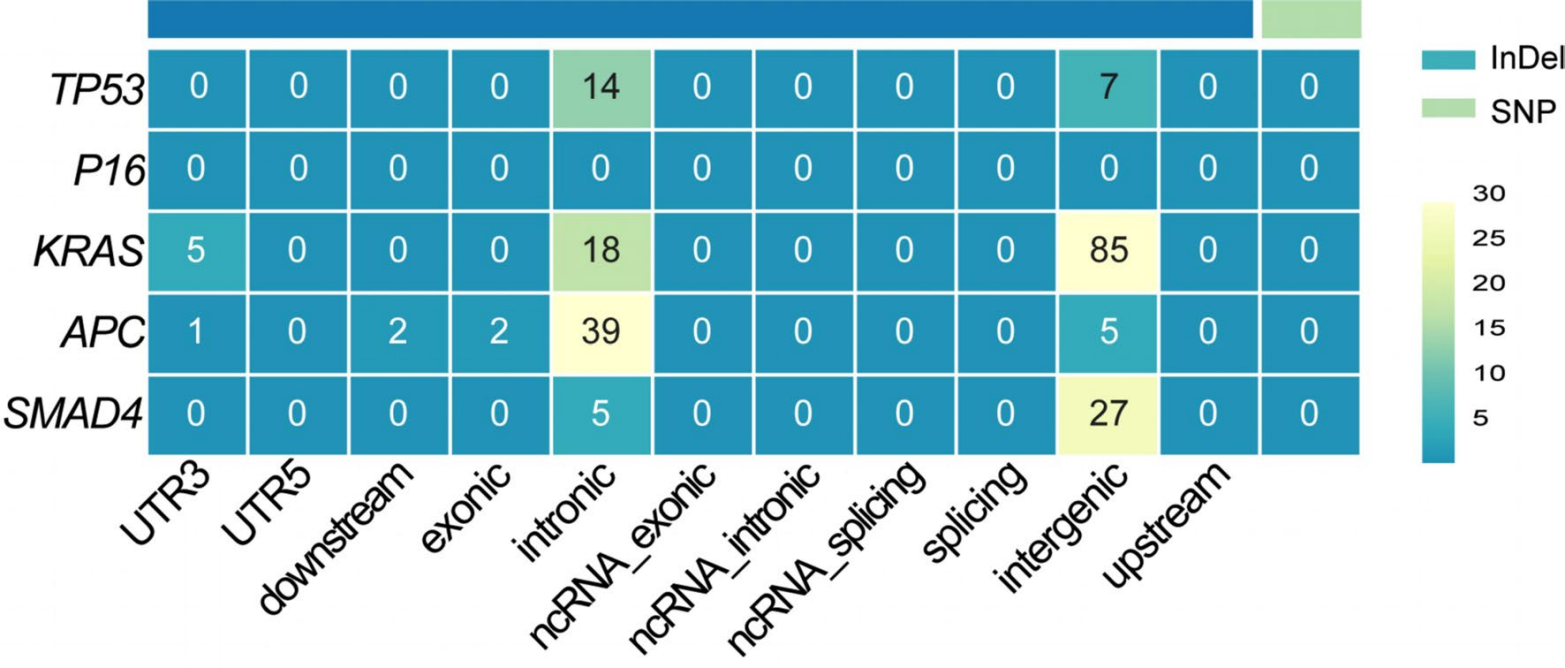
Distribution of InDel and SNP mutations. InDel refers to insertion or deletion mutations. SNP stands for single nucleotide polymorphism. *Exonic* denotes mutations occurring within the coding regions of the coding sequence (CDS). *Intronic* refers to mutations located in the intronic regions of genes. *Intergenic* pertains to mutations found in the spacer regions between genes. *UTR3* and *UTR5* represent mutations within the 3’ and 5’ untranslated regions (UTRs) of the gene, respectively. *Downstream* indicates mutations occurring within 2 kb downstream of the transcription start site. *Upstream* refers to mutations located within 2 kb upstream of the transcription start site

Whole-genome resequencing was conducted to identify mutations in *TP53*,* P16*,* KRAS*,* APC*, and *SMAD4* in DPC-X3 cells. The results are presented in Fig. 8. No single nucleotide polymorphisms (SNPs) were detected in these genes. However, *TP53* harbored 21 insertions and deletions (InDels), with 14 mutations located in intronic regions and 7 in intergenic regions. No mutations were identified in the *P16* gene. In *KRAS*, 108 InDels were observed, including 5 within the 3’ untranslated region (UTR), 18 in intronic regions, and 85 in intergenic regions. *APC* exhibited 49 InDels, of which 1 was in the 3’UTR, 2 within 2 kb downstream of the transcription start site, 2 within an exonic region, 39 in intronic regions, and 5 in intergenic regions. *SMAD4* harbored 32 InDels, with 5 in intronic regions, and 27 in intergenic regions.

## Discussion

Intestinal-type ampullary cancer typically manifests with a non-invasive duodenal adenoma component. Its subtype shares similarities with colorectal cancer, displaying central necrosis, cribriform, or tubular glands [21, 22]. Intestinal-type ampullary cancer exhibits a more limited invasive component and less common perineural and lymphovascular infiltration, contributing to a comparatively better prognosis than that of pancreaticobiliary-type AC [23, 24].

The scarcity of ampullary cancer CLs severely limits understanding its pathogenesis and drug development. Determining the optimal treatment regimen, particularly for supplementary treatment and drug-based interventions in progressed instances, is challenging. There is an urgent need to comprehend the mechanisms driving ampullary cancer occurrence and progression to explore effective treatments, ultimately prolonging patient survival [8, 25].

AC classification predominantly relies on morphological assessment through H&E staining, supplemented by IHC staining results of related proteins. When discrepancies arise between morphological evaluation and IHC examination, priority should be given to the former [3]. The primary tumor of DPC-X3 has a tissue structure that conforms to the characteristics of typical intestinal-type AC, and IHC results exhibited positive CK7 and CK20 expression in DPC-X3 cells, all these signifying DPC-X3 as an intestinal-type AC CL. This CL offers a platform for basic research and drug development on ampullary cancer.

Tumor cell resistance significantly contributes to treatment failures, posing a major challenge in cancer therapy. Hence, investigating the causes of tumor cell resistance and strategies to enhance their responsiveness to chemotherapy drugs is a current, complex research focus. Drug-resistant CLs are vital models for exploring drug resistance mechanisms in tumor cells [26–28]. Newly established CLs closely maintain primary tumor characteristics, providing research outcomes closest to real-world human scenarios [29–31]. DPC-X3 cells, identified with multidrug resistance, represent an intrinsic multidrug-resistant CL, making them an excellent model to investigate drug resistance mechanisms in AC. Understanding the resistance mechanism of DPC-X3 cells is anticipated to aid in the diagnosis, treatment, and drug development for intestinal ampullary cancer.

The abnormal chromosome count in cancer cells has been strongly associated with unfavorable patient outcomes [32, 33]. Recent research suggests that triploidy might correlate with innate drug resistance in tumors, whereas those with more than four sets are linked to developed drug resistance [34]. The karyotype of DPC-X3 cells is notably complex, with approximately 20% of cells being subtriploid. This complexity might contribute to the inherent multidrug resistance observed in DPC-X3.

EMT represents a transformation where epithelial cells gain mesenchymal traits, a phenomenon associated with tumor resistance [35–39]. EMT typically involves a diminish in E-cadherin, and an increase in Vimentin expression typically occurs. Alterations in gene expression during EMT lead to various phenotypic shifts, including changes in cellular morphology, diminished adhesion, and acquisition of stem cell–like properties. Crucial signaling cascades implicated in EMT encompass transforming growth factor-β, Wnt, Notch, and hedgehog pathways [40–42]. IHC examination revealed reduced E-cadherin expression and heightened Vimentin expression in DPC-X3 cells, indicating EMT characteristics, which likely contribute significantly to its inherent multidrug resistance.

The mouse transplant model holds immense value and has been pivotal in our in vivo research on tumor mechanisms, development, and drug testing [43, 44]. DPC-X3 cells inoculated subcutaneously into NXG mice result in swift and uniform establishment of transplanted tumors with a brief latency interval and a 100% formation frequency. Pathological examination revealed similarities between the histological characteristics of DPC-X3 grafts and those of primary tumors. These findings underscore the DPC-X3 transplantation tumor as an exceptional in vivo experimental prototype.

The main limitation of this investigation is the lack of comparison between DPC-X3 and SNU869, mainly because the type classification of SNU869 is not so certain. SNU869 was reported by Ku JL in 2002 [45], but H&E results of the original tissue were not reported in that article; in 2016, Lai et al. determined SNU869 as an intestinal-type AC CL through IHC [11], but the type classification of AC predominantly relies on morphological assessment through H&E staining. Based on this information, comparing these two CLs has limited significance. Fortunately, our team is about to establish another intestinal-type AC CL. Next, our group will conduct further research based on these established and ongoing CLs. This study mainly conducted preliminary verification and characterization of DPC-X3 at the macro level. In the future, our group will conduct in-depth molecular research based on the established AC CLs to further reveal the development mechanism of AC and prevention and treatment methods for this cancer.

In summary, this study introduced a novel intestinal-type AC CL, DPC-X3, derived from a Chinese man. This newly established CL offers a promising and robust experimental model for fundamental research and drug development in the context of intestinal-type ampullary cancer.

## Data Availability

The datasets generated and/or analysed during the current study are available in the NCBI repository. Accession to cite for these SRA data: PRJNA1194522. Submission ID: SUB14910632. Databank URL: https://www.ncbi.nlm.nih.gov/sra/PRJNA1194522.
